# Evaluating the predictive effect of vitamin D on clinical outcomes of infliximab-treated Crohn’s disease patients

**DOI:** 10.3389/fimmu.2025.1578191

**Published:** 2025-06-04

**Authors:** Jiao Zheng, Hongping Gao, Yu Zhang, Mingxuan Sun, Hang Sun, Ying Pei

**Affiliations:** ^1^ Department of Anorectal, Affiliated Municipal Hospital of Xuzhou Medical University, Xuzhou, Jiangsu, China; ^2^ Department of Endocrinology, Xuzhou Municipal Hospital Affiliated to Xuzhou Medical University, Xuzhou, Jiangsu, China

**Keywords:** Crohn’s disease, vitamin D, infliximab, remission rate, gastroenterology

## Abstract

**Backgrounds:**

The aim of this study was to examine the clinical predictors of Infliximab (IFX) in Crohn’s disease (CD) patients in eastern China and to support further research on the vitamin D remission rate compared to CD patients.

**Methods:**

Patients with CD who were hospitalized at Xuzhou First People’s Hospital between January 2020 and December 2023 were included in our retrospective analysis. Clinical information was gathered from CD patients at baseline and the endpoint (7th IFX therapy, 38 weeks). To determine the baseline variable [Crohn’s Disease Activity Index (CDAI) < 150] for endpoint clinical remission in patients receiving IFX, and to examine the relationship between blood vitamin D (VIT-D) levels before starting IFX medication and CDAI at Week 38. The potential risk variables were then investigated using univariate, multivariate, and LASSO regression models.

**Results:**

Included were 158 individuals with CD treated with IFX. At baseline, 18.35% of patients had a VIT-D deficit; 64.19% of patients experienced a decrease in VIT-D, and 63.29% of patients achieved clinical remission. The high Vitamin D levels at baseline were independent predictors of clinical remission after IFX therapy, according to univariate, multivariate, and LASSO regression analysis (P < 0.05). Receiver operating characteristic curve analysis revealed that AUC (95%CI) 0.56(0.25-0.95) was the endpoint CDAI (= 150) diagnostic value when the Vit-D level was 19.35 ng/ml. The corresponding sensitivity and specificity were 75.02% and 79.6%. Endpoint CDAI was independently predicted by male sex, age, BMI, and VIT-D levels <30 ng/ml (P < 0.05).

**Conclusion:**

After receiving IFX therapy, CD patients in eastern China with higher VIT-D levels were more likely to achieve clinical remission, particularly those who were male, older, had a higher BMI, and had VIT-D levels below 30 ng/ml.

## Introduction

Crohn’s disease, an inflammatory condition of the gut driven by a dysregulated immune response, is one of many chronic inflammatory disorders affecting the gastrointestinal tract (1). In Asia, China reports the highest rate of CD diagnoses (3.44 per 100,000) (2). Current therapeutic goals primarily focus on promoting mucosal healing to slow disease progression (3). The complete development of the illness is likely influenced by a complex interplay of infectious and environmental factors (4). Notably, CD is associated with low levels of sunshine exposure, and both forms of CD are more common in North America and Europe as latitude increases (5). In CD, biologics targeting tumor necrosis factor-α (TNF-α) significantly increase response rates and rates of clinical response. However, clinical relapse and disease progression may result from a loss of response to anti-TNF-α therapy over time (6). It is estimated that 30% of inflammatory bowel disease (IBD) patients experience a loss of response to infliximab (IFX) during the course of therapy (7). Therefore, enhancing the efficacy and responsiveness to anti-TNFα agents is crucial.

Vitamin D3 is a nutrient essential for healthy bone production and growth (3). V Vitamin D can modulate both innate and adaptive immune responses (8). Vitamin D deficiency is associated with increased autoimmunity and heightened susceptibility to infection (9). Since immune cells in autoimmune diseases respond to the ameliorating effects of vitamin D, the benefits of supplementation in individuals with vitamin D deficiency may outweigh those on bone and calcium homeostasis (10). Vitamin D has emerged as a key regulator of the innate immune response to pathogen threat (11, 12). The hormone form of vitamin D signals through nuclear receptor transcription factors and regulates gene transcription (3). The link between vitamin D deficiency and CD is also gaining traction (1). The incidence of inflammatory bowel disease (IBD) is linked to vitamin D insufficiency, regardless of whether it results from inadequate dietary intake or a lack of sun exposure and skin synthesis (13). Vitamin D deficiency has been shown to worsen colitis progression in animal models, and vitamin D supplementation has been shown to restore epithelial integrity and inhibit inflammatory cytokine levels, thereby improving intestinal inflammation (3). Recent research has also shown that vitamin D levels in CD patients treated with IFX tend to correlate with IFX plasma concentrations. Growing evidence suggests that vitamin D plays a crucial role in regulating both innate and adaptive immunity, and could serve as a predictive factor for CD patients (1, 14). It remains unclear, however, how vitamin D functions in CD and what specific indicators may predict clinical outcomes.

While the relationship between vitamin D and IFX-treated CD disease has been reported in western China, its role in eastern China remains less understood (15). In order to determine the relationship between blood vitamin D levels and clinical remission in patients treated with CD by IFX, we first examined the clinical baseline data of CD patients treated with IFX.

## Materials and methods

According to the inclusion and exclusion criteria listed below, clinical data from patients in the Crohn’s disease patient database in the Department of Gastroenterology at Xuzhou First People’s Hospital was gathered for this study using a retrospective survey method between January 2020 and December 2023. The Xuzhou City First People’s Medical Ethics Committee gave its approval to this research.

### Inclusion criteria

The study’s inclusion criteria were as follows: 1) CD patients diagnosed using Chinese diagnostic criteria for inflammatory bowel disease (16); 2) aged between 18 and 60 years; 3) receiving Infliximab (IFX) for their first course of therapy; 4) having baseline 25-hydroxyvitamin D (25(OH)D) levels recorded before initiating IFX medication; and 5) having a recorded Crohn’s Disease Activity Index (CDAI) score at the endpoint (Week 38), following the seventh IFX treatment. Exclusion criteria included: 1) individuals lacking routine follow-up; 2) individuals treated with IFX less than three months before the study start date; 3) patients with a history of glucocorticoid use within three months before initiating IFX medication; and 4) patients with severe or chronic cardiovascular, pulmonary, urinary, endocrine, reproductive, skeletal, muscular, neurological, or other systemic disorders, or those with other active infectious diseases.

### Outcome

Prior to stratifying by vitamin D status, we initially examined baseline data from CD patients receiving IFX (17). According to the Endocrine Society Clinical Practice Guidelines 2011, patients with 25(OH)D levels < 15 ng/mL were classified as deficient, those with levels > 30 ng/mL as sufficient, and those with levels between 15 and 30 ng/mL as insufficient (18).

### Clinical, and biological assessment

IFX therapeutic doses were administered ranging from 5 to 10 mg/kg as specified by IFX guidelines (100 mg/infusion). Treatment intervals followed standard protocols: weeks 0, 2, and 6 for the induction phase and every 8 weeks thereafter for the maintenance phase. At Week 38, clinical outcomes were evaluated, including clinical remission, biochemical remission, endoscopic remission, clinical response, and endoscopic response. Patients were classified into remission and non-remission groups based on clinical remission criteria. Subsequently, the association between baseline characteristics, Time-Drug Exposure (TDM), Area Under the Curve for Drug Concentration (ATI), and clinical remission status was analyzed.

Baseline clinical data collected for CD patients included disease course, symptoms, history of bowel and perianal surgery, use of combined immunosuppressants (IMM), Montreal classification, CDAI score, Simple Endoscopic Score for Crohn’s Disease (SES-CD), inflammatory markers, nutritional indicators, liver and kidney function, electrolytes, fasting blood glucose (FBG), blood lipids, and antinuclear antibodies (ANA). Clinical remission was defined as a CDAI score < 150, clinical response as a CDAI reduction ≥ 70 (baseline to Week 7), biochemical remission as a C-reactive protein (CRP) < 5 mg/L, endoscopic remission as SES-CD ≤ 4, and endoscopic response as a ≥ 50% reduction in SES-CD from baseline to Week 38 (19, 20). Patients meeting these remission criteria could be considered for discharge. The IFX dosage (ranging from 5 to 10 mg/kg) adhered to the specified infusion dose (100 mg) and standard protocols for induction (weeks 0, 2, 6) and maintenance (every 8 weeks).

### Prognostic nomogram analysis

The LASSO (Least Absolute Shrinkage and Selection Operator) method was used to identify and select risk factors from the multivariate data. Variables with non-zero LASSO regression coefficients were selected for further analysis. Multiple logistic regression analysis was performed on these selected variables within the LASSO regression model to construct a predictive model. A calibration curve was created to assess the nomogram’s calibration, and the Harrell c-index was computed to measure its discriminative power. Decision curve analysis was performed to evaluate the nomogram’s clinical utility across different threshold probabilities and to assess net benefit. The diagnostic efficacy of the clinical factor model was assessed using the Receiver Operating Characteristic (ROC) curve (AUC) from the ROC toolbox (21).

### Statistical analysis

The statistical program SPSS version 26.0 was used to analyze the data. Normality of continuous variables was tested using the Shapiro-Wilk test. Data normally distributed are presented as mean ± standard deviation (SD); comparisons between groups for normally distributed data were conducted using independent-sample t-tests. Data not normally distributed are presented as median (Q1, Q3); comparisons between groups for non-normally distributed data were conducted using nonparametric tests. Categorical data are presented as counts (n) and percentages (%). Each variable underwent univariate analysis. Variables with a P-value < 0.1 from univariate analysis were included as candidates for multivariate logistic regression analysis. The threshold for statistical significance was set at P < 0.05. Subgroup analyses were conducted to investigate the variability of treatment effects and influencing variables among different patient groups. ROC curve analyses were performed comparing the endpoint CDAI results with the aforementioned subgroup factors. A P-value < 0.05 in the final multivariate model was considered significant.

## Results

### Baseline characteristic

Ten of the 185 patients we recruited were disqualified because there was insufficient information on vitamin D insufficiency, as **Table 1** illustrates. Furthermore, the absence of follow-up data led to the exclusion of 17 individuals. Thus, there were 158 CD patients hospitalized in all (**Supplementary Figure 1**). The patients were 36.95 ± 3.65 years old on average. There are seventy-five ladies. There were 87 patients with prior surgery and 45 smokers, with a BMI score of 22.15 ± 2.48.

**Table 1:** Baseline patient characteristics.

Characteristic	All, N=158	Serum vitamin D			
		<15 ng/mL, N = 29 (18.35%)	15–30 ng/mL, N = 103(65.19%)	>30 ng/mL, N = 26 (16.46%)	p value
Age, years (mean ± SD)	36.95 ± 3.65	32.48 ± 4.95	37.15 ± 6.25	35.66 ± 3.91	0.28
Gender, female, n (%)	75	15	55	5	0.44
Disease duration, year(mean ± SD)	8.29 ± 1.62	5.61 ± 0.95	9.86 ± 2.61	9.34 ± 1.05	0.39
Disease location, n (%)					
Ileum	45	10	28	7	0.05
Colon	30	2	25	3	
Ileum-colon	61	10	38	13	
Upper GI	22	7	12	3	
Disease behavior, n (%)					
Inflammatory	36	12	20	4	0.58
Stricturing	48	9	30	9	
Penetrating	74	8	53	13	
Perianal disease, n (%)	45	9	31	5	0.55
Current smoker, n (%)	28	11	14	3	0.15
Previous use of anti-TNF-α agents, n (%)	24	7	10	7	0.25
Naïve to biologics, n (%)	31	9	12	10	0.68
History of surgery, n (%)	87	12	62	13	0.24
Previous surgery, median (IQR), n	3	1	1	1	0.09
Type of surgery, n (%)					
Small bowel resection	45	12	29	4	0.24
Colon resection	63	12	41	10	
Ileocecectomy or ileocolectomy	50	5	33	12	
Postoperative medication, n (%)					
No medication	42	6	30	6	0.25
Metronidazole	27	3	20	4	0.11
Steroid	28	5	21	2	0.24
Immunomodulators	75	24	38	13	0.62
Biologic use, n (%)					
No biologic	71	14	50	7	0.11
Anti-TNF-α agents	52	8	30	14	
Vedolizumab	35	7	23	5	
Vitamin D supplement, n (%)	62	7	49	6	0.01
Vitamin B12, pg/mL (IQR)	561.25 ± 12.62	385.48 ± 15.94	482.61 ± 21.58	594.55 ± 21.05	0.2
Zinc, μg/mL (IQR)	0.78 ± 0.08	0.61 ± 0.08	0.67 ± 0.11	0.72 ± 0.03	0.16

GI, gastrointestinal tract; IQR, interquartile-range.

Second, we divided the groups based on vitamin D levels, and we discovered that 29 and 103 individuals, respectively, were in the vitamin D deficient group. Diarrhea and stomach discomfort were the primary clinical signs. Age, sex, ethnicity, disease course, history of prior surgery, perianal disease, intestinal length resection, vitamin B12 or zinc levels, history of anti-TNF use, and the site and behavior of the disease (inflammation, stricture, or penetration) did not differ among the three groups.

The highest tritile of vitamin D had the greatest percentage of patients using supplements (p = 0.02). We were unable to statistically examine dosage associations since most patients were using over-the-counter vitamin D pills. The usage of biologics and postoperative immunomodulators did not significantly vary across groups.

### Change from baseline data for clinical remission

The CD patients were separated into two groups, one for clinical remission (100 instances) and the other for clinical non-remission (58 cases), based on their response status after IFX therapy (CDO score <150), as shown in **Table 2**. We discovered that the non-remission group’s levels of vitamin D, BUN, and Cr were significantly lower than those of the remission group. Second, we discovered that compared to the remission group, the non-remission group had noticeably greater levels of serum ferroglobin, ATI, HS-CRP, ESR, and CRP. Hemoglobin, white blood cell, and liver function indices did not change significantly.

**Table 2:** Analysis of differences in clinical factors between the two groups.

Analyte	Remission group (n = 100)	Non-remission group (n = 58)	OR	95% CI	P
Vit-D	20.98 ± 6.93	18.23 ± 6.99	0.89	0.32-0.89	0.012
CRP	12.34 ± 3.21	78.20 ± 9.32	1.23	0.55-1.83	0.011
ESR	18.22 ± 2.34	46.22 ± 5.21	1.2	0.99-1.92	0.032
Hs-CRP	28.93 ± 5.93	72.92 ± 6.11	2.34	1.42-3.23	0.016
WBC	6.03 ± 1.02	8.93 ± 1.42	1.11	0.63-1.63	0.52
Hb	129.52 ± 21.84	89.25 ± 25.31	2.93	1.23-4.22	0.113
ALB	42.61 ± 3.68	34.69 ± 4.69	1.05	0.68-1.58	0.512
AST	14.78 ± 5.62	12.34 ± 2.11	0.98	0.42-1.56	0.582
ALT	11.62 ± 1.36	8.26 ± 1.26	0.68	0.31-1.11	0.625
ALP	75.64 ± 6.35	74.95 ± 6.89	6.84	2.15-9.65	0.381
TBil	9.35 ± 0.95	6.42 ± 0.69	1.05	0.34-1.66	0.582
BUN	4.39 ± 0.95	3.05 ± 0.65	0.86	0.44-1.25	0.025
Cr	68.27 ± 12.65	60.11 ± 11.02	0.68	0.42-0.99	0.045
Uric Acid	352.61 ± 85.64	301.08 ± 78.95	1.36	0.89-1.99	0.485
VB12	443.95 ± 25.61	482.61 ± 23.68	1.68	0.89-1.99	0.586
IFX-TC	4.25 ± 0.86	2.03 ± 0.98	0.56	0.14-0.99	0.066
ATI, n (%)	8	14	1.02	0.85-1.68	0.002
Blood glucose	4.36 ± 0.95	4.29 ± 0.68	1.25	0.69-1.99	0.625
Serum Ferritin	78.69 ± 10.25	199.25 ± 19.64	0.89	0.48-1.52	0.032

vit-d, Vitamin D; CRP,C-reactive protein; ESR, Erythrocyte sedimentation rate; Hs-CRP, Highly sensitive C-reactive protein; WBC, White blood cell; Hb: hemoprotein; ALB, albumin; AST, Aspartate aminotransferase; ALT, glutamic-pyruvic transaminase; ALP, alkaline phosphatase; TBil, Total Bilirubin; BUN, Urea nitrogen; Cr, creatinine; VB12, Vitamin B12; IFX-TC, infliximab trough concentration, ATI, anti-TNFα antibody; OR, odds ratio.

### Univariate and multivariate analyses influenced the assessment of risk factors

As indicated in **Table 3**, univariate analysis revealed a strong correlation between poor clinical remission following IFX treatment and age, vitamin D3 levels significantly below 30 ng, and vitamin D supplementation. Subsequent multivariate analysis revealed a strong correlation between poor clinical remission following IFX treatment and age and vitamin D deficiency.

**Table 3:** Factors assessed with univariate and multivariate analysis.

Factors	Univariate			Multivariate		
	OR	95% CI	p value	OR	95% CI	p value
Age (>35)	2.68	0.85-6.25	0.01	1.18	0.32-2.61	0.03
Disease vitamin D triplet (>6 years)	1.22	0.25-3.69	0.28	2.68	0.96-3.62	0.09
Female	2.95	0.24-6.35	0.28	1.65	0.88-2.68	0.06
Perianal disease	0.35	0.02-4.62	0.95	2.69	1.25-3.66	0.11
Anti-TNF naïve	0.58	0.24-5.62	0.21	1.69	0.36-2.68	0.36
Vitamin D <30 ng/mL	0.36	0.08-1.25	0.02	0.89	0.42-2.26	0.03
Vitamin D supplement	0.22	0.18-0.95	0.09	2.69	0.98-4.85	0.12
Vitamin B12 >240 pg/mL	1.25	0.65-3.69	0.55	0.89	0.21-1.25	0.09
Zinc >0.68 μg/mL	1.69	0.98-2.26	0.11	2.61	1.25-4.58	0.16

OR, odds ratio.

A ROC curve analysis was conducted between the endpoint CDAI results and the aforementioned subgroup factors. AUC (95%CI) 0.56(0.25-0.95) was the endpoint CDAI (= 150) diagnostic value in the male subgroup when the Vit-D level was 19.35 ng/ml. The sensitivity and specificity were 0.75 and 0.79 (P = 0.015), respectively, suggesting a greater impact on normal-weight persons. The endpoint CDAI (= 150) diagnostic value was AUC (95%CI) 0.78 (0.68-0.89) in the normal BMI subgroup when the Vit-D level was 16.25 ng/ml. The sensitivity and specificity were 0.68 and 0.86 (P = 0.032), respectively, suggesting a greater impact on normal-weight people. When the Vit-D levels were 19.45 ng/ml, the diagnostic value of the endpoint CDAI (= 150) in the smoke-free subgroup was AUC (95% CI) 0.77 (0.61-0.85), the sensitivity was 0.85, and the specificity was 0.88 (P = 0.024). Consequently, vitamin D supplementation may be especially beneficial for this subgroup in the treatment of Crohn’s disease (**Table 4**).

**Table 4:** Diagnostic efficiency of different subgroups for endpoint CDAI outcomes by ROC curve.

Subgroup	Diagnostic value (Vit-D baseline level ng/ml)	AUC(95%CI)	Sensitivity	Specificity	P
Male	19.35	0.56(0.25-0.95)	0.75	0.79	0.015
BMI: normal weight	16.25	0.78(0.68-0.89)	0.68	0.86	0.032
No smoking	19.45	0.77(0.61-0.85)	0.85	0.88	0.024
Disease duration (>6 years)	18.22	0.78(0.65-0.85)	0.78	0.77	0.158
without Perianal disease	16.35	0.77(0.58-0.85)	0.85	0.81	0.248
Without intestinal surgery	15.64	0.84(0.55-0.95)	0.79	0.62	0.258

CDAI, Crohn's Disease Activity Index; ROC, receiver operating characteristic; BMI, Body Mass Index; AUC, Area under the curve.

### LASSO regression and nomogram validation

**Figure 1:**
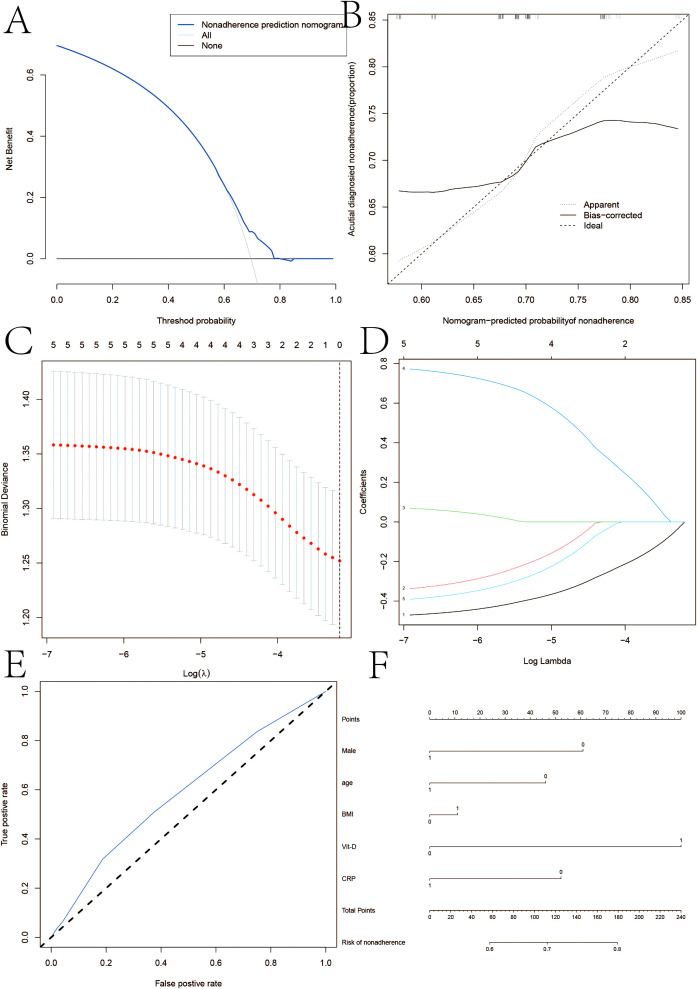
**(A)** Calibration curves of the nonadherence nomogram prediction in the cohort. **(B, C)** Decision curve analysis for the nonadherence nomogram and AUC curve. **(D)** LASSO coefficient profiles of the 20 characteristics. A coefficient profile plot was constructed against the log(lambda) sequence. **(E, F)** ertical line was drawn at the value determined using fivefold cross-validation, where optimum lambda resulted in five features with nonzero coefficients in iron homeostasis in SSNHL patients.

Multiple linear regression models demonstrated a connection between Crohn’s disease and vitamin D, as seen in **Figure 1**. In multivariate logistic regression models, Crohn’s disease was also independently predicted by male, age, BMI, and vitamin D levels <30 ng/ml (**Table 3**). Fifteen variables were narrowed down to five potential predictors with nonzero coefficients in the LASSO regression model based on the 158 patients in the cohort (4:1 ratio). These included being male, being older, having a BMI, and having vitamin D levels below 30 ng/ml. The model that included the previously described independent predictors was used to produce the nomogram. The nonadherence risk nomogram’s calibration curve for risk prediction in Crohn’s disease, meantime, showed high consistency in this population. The model demonstrated high discrimination, as shown by the C-index for the cohort’s prediction nomogram, which was 0.982 (95 percent CI, 0.25–1.03) and was confirmed to be 0.925 after bootstrapping validation. The decision curve analysis for the vit-D-linked indicators nomogram is shown in **Figure 1B**, and the area under the curve (AUC) was 0.968, according to **Figure 1C**. In summary, a vitamin D deficit might be a useful predictor of Crohn’s disease outcome.

## Discussion

Human health is significantly impacted by the high frequency and poor prognostic outcomes of CD patients (22, 23). Anti-TNF treatment for CD patients, however, has limited remission impact, making improving the treatment’s remission rate a crucial area of study (24, 25). Furthermore, prevalent vitamin D deficiency is observed in CD patients, with over 50% affected. Comparisons of baseline data revealed that the non-remission group had a significantly higher rate of vitamin D deficiency. Subsequent studies utilizing univariate, multivariate, and LASSO regression models further identified vitamin D insufficiency as a significant risk factor for CD patients receiving IFX intervention.

In 2006, Robert Modlin’s group demonstrated that stimulating macrophages through the pattern recognition receptor toll-like receptor 2 (TLR2) results in the expression of CYP27B1, leading to endogenous production of 25-hydroxyvitamin D (25(OH)D) (26, 27). T This finding is considered a key mechanism supporting the role of 1,25-dihydroxyvitamin D (1,25D) signaling in the innate immune response. For many years, it has been recognized that vitamin D deficiency is associated with CD.

Schäffler et al. found that CD patients benefited from therapy with tumor necrosis factor-α inhibitor and exhibited significantly higher vitamin D levels than those without (28). Shema Ayadi et al. also found that Vitamin D deficiency is common in Tunisian CD patients as well as in controls and is associated with disease activity. This association is often attributed to either intestinal malabsorption of dietary vitamin D or insufficient sun exposure during active illness, particularly in high-latitude populations (1). Nonetheless, evidence from the aforementioned laboratory research strongly suggests that vitamin D insufficiency may play a role in the pathophysiology of CD, and that sufficient vitamin D supplementation may improve innate immunity, reduce inflammation, and alleviate CD symptoms. Numerous clinical trials conducted since 2010 have shown the potential therapeutic effects of potent vitamin D supplementation for CD patients. For example, a 2011 clinical study reported that women with anticipated 25(OH)D levels of >30 ng/mL (75 nM) had a multivariate-adjusted hazard ratio of 0.38 (95% CI, 0.15–0.97) compared to women with expected levels of <20 ng/mL (50 nM), suggesting that higher circulating 25(OH)D levels significantly lower the incidence of CD in this female population (29).

Vitamin D insufficiency is prevalent in the CD patient population and is independently linked to worse health-related quality of life (HRQOL) and increased disease activity, according to a retrospective cohort analysis of 403 CD patients (30). Furthermore, a retrospective analysis of pediatric populations revealed similar findings; 47% of children had vitamin D deficiency or insufficiency, and in this group, vitamin D deficiency was linked to higher exposure to corticosteroids (31, 32). Serum vitamin D levels were found to be substantially lower in children with CD compared to healthy controls, and the group of children recently diagnosed with IBD also exhibited high rates of vitamin D insufficiency or deficiency (31, 32). A 25(OH)D level of <20 ng/mL (50 nM) was linked to a statistically significant increase in the risk of surgery and hospitalizations due to IBD compared to individuals with levels >30 ng/mL, according to a multivariate study of over 1500 CD patients (33). In clinical research, many studies have reported the relationship between 25(OH)D levels and clinical outcomes in CD patients, as well as the effect of biologic agents (34). The lack or insufficiency of 25(OH)D is more common in CD patients. Furthermore, patients with low 25(OH)D levels are less likely to achieve clinical remission and a poorer response to biologic agents (35, 36). In a small, double-blind, randomized, placebo-controlled trial involving 27 CD patients in remission, those assigned to vitamin D (2000 IU/d) achieved a significantly higher 25(OH)D level (at least 75 nmol/L) compared to the placebo group, although the Crohn’s Disease Activity Index (CDAI) scores did not significantly decrease (37).

Only one study in western China examined the therapeutic effects of vitamin D combined with IFX. This study found that vitamin D levels in the subgroup of patients with a normal BMI, non-smoking status, and receiving immunosuppressant therapy had independent predictive value for the endpoint CDAI score (P < 0.05). This represents one of the relatively few studies on this specific topic. Following IFX medication, baseline vitamin D levels in CD patients—particularly those with a normal BMI, who do not smoke, and who receive IFX in addition to immunosuppressants—predict clinical remission (15). However, this research focused on the examination of vitamin D levels and IFX-induced remission in CD patients within an eastern region, likely due to significant differences in latitude and altitude between the eastern and western parts of China.

Potential underlying mechanisms linking the effectiveness of IFX and vitamin D therapy may include inhibiting VDR expression and function, increasing downstream inflammatory signaling, and enhancing the production of inflammatory cytokines (38). Some research suggests that intestinal epithelial integrity depends on Vitamin D and that these cells are closely linked to VDR expression. In experimental colitis models using VDR knockout (VDR-/-) mice, animals exhibit increased vulnerability to epithelial damage, typically characterized by disruption of epithelial integrity and loss of tight junctions, which consequently increases susceptibility to bacterial translocation (39–41).

Clinical investigations frequently demonstrate strong correlations among Vitamin D levels, clinical outcomes in CD patients, and the effects of biologics (34). Vitamin D deficiency or insufficiency is more prevalent in CD patients. Furthermore, patients with low Vitamin D levels often experience poor responses to biologics and have lower rates of clinical remission. In individuals with CD, Vitamin D status may even serve as a predictor of the long-term effectiveness of biologics, and supplementing these patients with Vitamin D can enhance biologic therapy (35, 36). Some research also indicates that individuals with Vitamin D insufficiency are more likely to discontinue IFX treatment due to poor response or the early emergence of anti-drug antibodies. Moreover, Vitamin D levels show a positive correlation with the duration of response to anti-TNF-α therapy (42).

### Limitations

A single-center retrospective research with a limited sample size is one of the study’s shortcomings. Another is that prospective intervention studies are required to further establish the causal association between vitamin D and clinical remission. We tried to use statistical methods to screen out the effect of each variable on the dependent variable after univariate analysis, taking into account several potentially significant variables. This was done to analyze the influencing factors more thoroughly and more in line with the actual clinical situation, given the bias of single-center, retrospective, and small-sample studies. To minimize bias from baseline values, we also used subgroup analyses at the same time. Second, information on vitamin D, calcium, and food consumption from sun exposure is lacking. Third, HPLC-MS produced more accurate findings than radioimmunoassay when it came to detecting VIT-D levels. However, only immunoassays may be carried out in our hospital laboratory.

## Conclusion

This research examined the association between vitamin D levels and CD patients in eastern China after IFX intervention. The findings showed a strong correlation between low vitamin D levels and the poor remission rate of CD patients following IFX intervention.

## Data Availability

The original contributions presented in the study are included in the article/**Supplementary Material**. Further inquiries can be directed to the corresponding author.

## Supplementary material

Supplementary Figure 1 Flow chart.
