# Correction: Evaluating the predictive effect of vitamin D on clinical outcomes of infliximab-treated Crohn’s disease patients

**DOI:** 10.3389/fimmu.2025.1651209

**Published:** 2025-07-15

**Authors:** Jiao Zheng, Hongping Gao, Yu Zhang, Mingxuan Sun, Hang Sun, Ying Pei

**Affiliations:** ^1^ Department of Anorectal, Affiliated Municipal Hospital of Xuzhou Medical University, Xuzhou, Jiangsu, China; ^2^ Department of Endocrinology, Xuzhou Municipal Hospital Affiliated to Xuzhou Medical University, Xuzhou, Jiangsu, China

**Keywords:** Crohn’s disease, vitamin D, infliximab, remission rate, gastroenterology

Affiliation “Department of Endocrinology, Xuzhou Municipal Hospital Affiliated to Xuzhou Medical University, Xuzhou, Jiangsu, China.” was erroneously omitted for author “Ying Pei”, and was incorrectly listed as “Department of Anorectal, Affiliated Municipal Hospital of Xuzhou Medical University, Xuzhou, Jiangsu, China”. This has been corrected to read:

“Jiao Zheng ^1^, Hongping Gao^1^, Yu Zhang^1^, Mingxuan Sun^1^, Hang Sun^1^, Ying Pei^2^.

1. Department of Anorectal, Affiliated Municipal Hospital of Xuzhou Medical University, Xuzhou, Jiangsu, China.

2. Department of Endocrinology, Xuzhou Municipal Hospital Affiliated to Xuzhou Medical University, Xuzhou, Jiangsu, China

The original version of this article has been updated.

