# Supplementary figures and images for: Evaluating the predictive effect of vitamin D on clinical outcomes of infliximab-treated Crohn’s disease patients
Source: Front Immunol. 2025 Jun 4;16:1578191. doi: 10.3389/fimmu.2025.1578191 (PMC12175088; doi:10.3389/fimmu.2025.1578191)

## CONSORT 2010 Flow Diagram

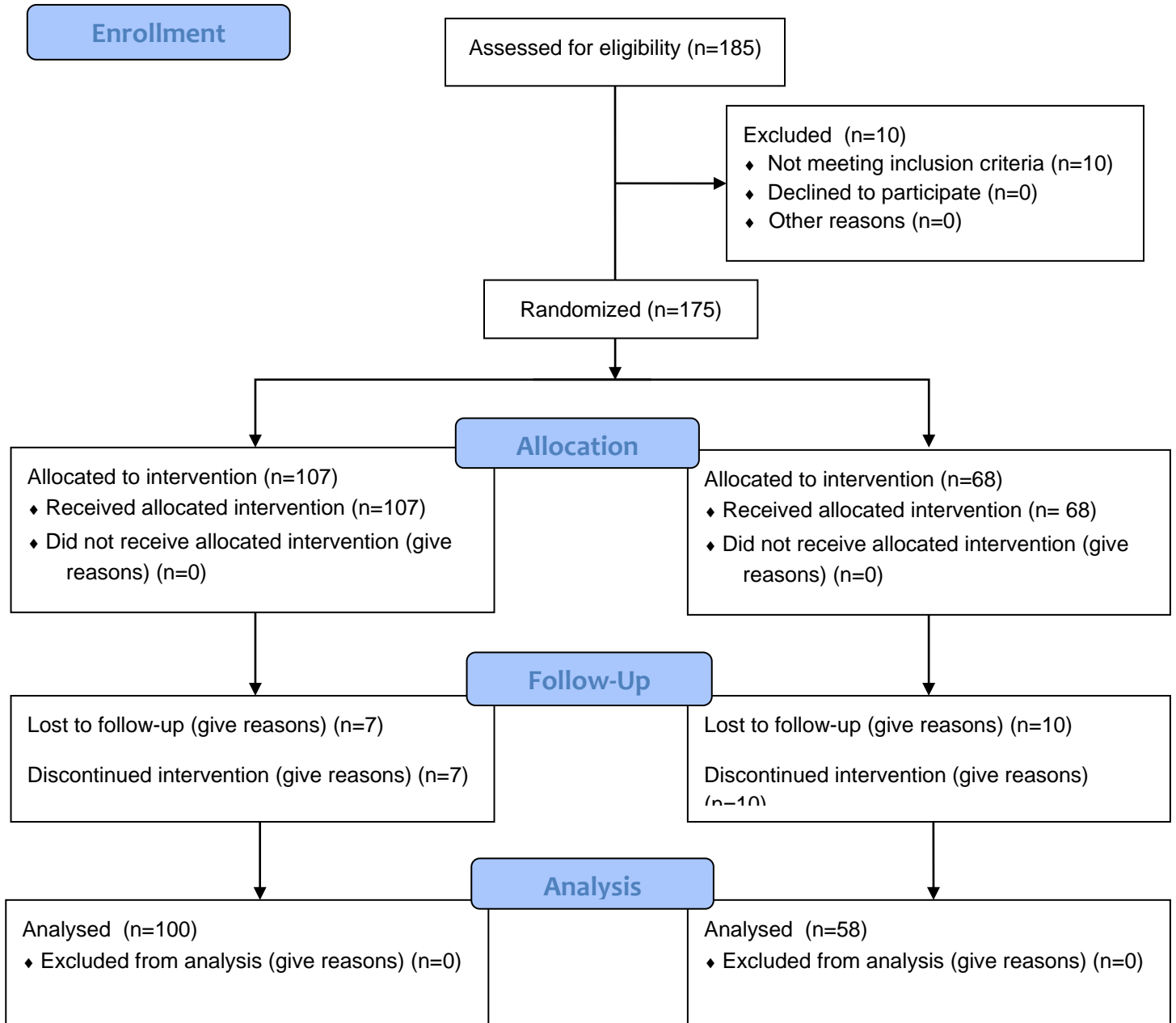

Supplement: Supplementary Figure 1 — Flow chart. [file Image1.pdf]
